# Longitudinal analysis of ear infection and hearing impairment: findings from 6-year prospective cohorts of Australian children

**DOI:** 10.1186/1471-2431-13-28

**Published:** 2013-02-21

**Authors:** Vasoontara Yiengprugsawan, Anthony Hogan, Lyndall Strazdins

**Affiliations:** 1 The Australian National University, National Centre for Epidemiology and Population Health, Acton, Canberra, ACT 2601, Australia; 2 The Australian National University, School of Sociology, Beryl Rawson Building, Acton, Canberra, ACT 2601, Australia

**Keywords:** Ear infection, Hearing impairment, Hearing loss, Longitudinal study, Australia

## Abstract

**Background:**

Middle ear infection is common in childhood. Despite its prevalence, there is little longitudinal evidence about the impact of ear infection, particularly its association to hearing loss. By using 6-year prospective data, we investigate the onset and impact over time of ear infection in Australian children.

**Methods:**

We analyse 4 waves of the Longitudinal Study of Australian Children (LSAC) survey collected in 2004, 2006, 2008, and 2010. There are two age cohorts in this study (B cohort aged 0/1 to 6/7 years N=4242 and K cohort aged 4/5 to 10/11 years N=4169). Exposure was parent-reported ear infection and outcome was parent-reported hearing problems. We modelled ear infection onset and subsequent impact on hearing using multivariate logistic regressions, reporting Adjusted Odds Ratios (AOR) and Confidence Intervals (95% CI). Separate analyses were reported for indigenous and non-indigenous children.

**Results:**

Associations of ear infections between waves were found to be very strong both among both indigenous and non-indigenous children in the two cohorts. Reported ear infections at earlier wave were also associated with hearing problems in subsequent wave. For example, reported ear infections at age 4/5 years among the K cohort were found to be predictors of hearing problems at age 8/9 years (AOR 4.0, 95% CI 2.2-7.3 among non-indigenous children and AOR 7.7 95% CI 1.0-59.4 among indigenous children). Number of repeated ear infections during the 6-year follow-up revealed strong dose–response relationships with subsequent hearing problems among non-indigenous children (AORs ranged from 4.4 to 31.7 in the B cohort and 4.4 to 51.0 in the K cohort) but not statistically significant among indigenous children partly due to small sample.

**Conclusions:**

This study revealed the longitudinal impact of ear infections on hearing problems in both indigenous and non-indigenous children. These findings highlight the need for special attention and follow-up on children with repeated ear infections.

## Background

Middle ear infection (otitis media) is a common childhood illness. Estimates indicate that by the age of 3 years at least half of children have experienced at least one episode of otitis [1-3]. Because it is a childhood illness, it requires close monitoring of signs and symptoms from parents and practitioners [4-6], and it is often co-morbid with other infections of the upper or lower respiratory tract [7,8].

One of the main consequences of otitis media is conductive hearing loss [9-11]. Hearing loss may result in speech and language disorders as well as a delay in academic development [12,13]. Subsequent behavioural problems from hearing impairment could also increase the risk of mental health disorders [14]. Otitis media affects children’s quality of life, and depending on the severity of infection, may also result in a wider impact on caregivers and health services [15,16].

In a comprehensive report on otitis media in Australia reported by Access Economics, the number of cases in 2008 were estimated to be at least 650,000 [17]. Treatment costs of visits to general practitioners and medical prescriptions were estimated between A$100 to A$400 million [18]. The cost of otitis media is more than $5 billion in the United States [19]. Reports on otitis media in children have shown that indigenous children are much more likely to be diagnosed with severe ear infection and to suffer repeated or multiple episodes than non-indigenous children [20-22]. Limited access to medical care, lower socioeconomic status, and remote living conditions all contributed to early childhood hearing loss among indigenous children [17,23].

However there is little longitudinal evidence which identifies the timing of otitis media onset and sequelae related to multiple or repeated infections. This paper examines the epidemiology of ear infection and subsequent hearing problems using unique 6-year prospective longitudinal Australian data in two cohorts of children (0/1 to 6/7 years and 4/5 to 10/11 years) including analyses for both indigenous and non-indigenous children.

## Methods

### Growing up in Australia, the longitudinal study of Australian children (LSAC)

The LSAC aims to identify the social, familial and individual factors influencing children in their early years of development, using a longitudinal study of two child age cohorts [24-26]. The study collected data from mothers, fathers, teachers, carers, as well as direct observations of children.

The LSAC used a two-stage clustered sampling design, stratified by state and by metropolitan/urban status. Children were randomly selected using the Medicare database. Detailed information on development of questions, study design, and non-response are reported in the literature [27,28]. Technical and discussion papers related to the project are also available to the public at http://www.aifs.gov.au/growingup. The author has obtained a license to use LSAC data approved by Department of Families, Housing, Community Services, and Indigenous Affairs (FaHCSIA) of the Australian Government.

The two child cohorts were recruited in 2004: those born between March 1999 and February 2000 (aged 4–5 year) and those born between March 2003 and February 2004 (aged 0–1 year). The interviews were conducted every two years (2006, 2008, and 2010) with between-wave mailout questionnaires. Compared to the Australian Bureau of Statistics population estimates, LSAC is broadly representative of the population, with some under-representation of families where parents have 12 years or less education, single-parent households, non-English speaking households, and families living in rental properties [28,29].

### Study population and statistical analyses

This study uses 4 waves of LSACs data currently available (2004, 2006, 2008, and 2010). There were 5,107 children at the baseline in the *Baby* or infant cohort (B cohort) at aged 3–19 months and 4,983 children at the baseline in the *Kindergarten* cohort (K cohort) aged 4 years 3 months to 5 years 7 months. At Wave 4 in 2010, there were 4,242 in the B cohort and 4,169 children in K cohort—83.1% and 83.7% response rate, respectively.

Sociodemographic characteristics of the sample available in this study included age and sex of parents and children in both cohorts, indigenous status of the study child, employment and school completion of parent, and remoteness. Socio-Economic Indexes for Areas (SEIFA), developed by the Australian Bureau of Statistics to rank area economic resources was included in the analyses [30].

Generally, the B and K cohort children were comparable on most socio-demographic characteristics, as shown in Table 1. Data on children were primarily collected via interview and questionnaire responses from the parent who knew the child best, mostly the child’s biological mother (over 97% for both cohorts). The mean age of the primary caregiver or parent was 31.0 years (SD ±5.5) in the B cohort and 34.7 years (SD ± 5.6) in the K cohort. Slightly more than half of children in both cohorts were males and the majority of children sampled spoke English as the main language at home. There was less than 5% of children who reported indigenous status in each cohort (230 children in the B cohort and 187 children in the K cohort). Parents in the B cohort were slightly better off socio-economically—66.7% reported school completion of year 12 and higher compared to 58.1% among the K cohort. Population and longitudinal sample weights were used in the analyses to correct for minor non-representativeness and attrition. The analyses were also adjusted for household clustering.

**Table 1:** Basic characteristic of the B and K cohort children

**Socio-demographic attributes**	**Percent (n)**	
	**B cohort 0/1 years**	**K cohort 4/5 years**
	**N=4,242**	**N=4,169**
Child characteristics		
Female	48.9 (2,497)	49.1 (2,446)
Indigenous status	4.5 (230)	3.8 (187)
English as main language at home	89.2 (4,555)	87.5 (4,359)
Parent characteristics		
Biological parent	99.7 (5,093)	99.4 (4,953)
Female	98.6 (5,033)	97.1 (4,839)
Mean age (± sd)	31.0 (±5.5)	34.7 (±5.5)
Parent 1 employment status		
Employed	49.7 (2,531)	57.4 (2,852)
Unemployed	3.2 (165)	3.8 (188)
Not in labour force	47.1 (2,400)	38.9 (1,932)
Parent 1 school completion		
Year 12+	66.7 (3,404)	58.1 (2,895)
Year 10/11	28.3 (1,443)	34.9 (1,739)
Year 9 or less	5.0 (256)	6.9 (344)
Remoteness area		
Highly accessible	54.8 (2,800)	53.8 (2,655)
Accessible	23.3 (1,188)	23.5 (1,159)
Moderately accessible	16.5 (840)	17.3 (855)
Remote/very remote	4.3 (221)	4.4 (217)
SEIFA economic resources		
Below median	47.3 (2,413)	45.8 (2,284)

Children’s *ear infection* (exposure variable) was assessed by parent (“Does child have any of these ongoing problems… ear infection (yes, no)?”). It is likely that the reported ear infections include acute otitis media, chronic suppurative otitis media, otitis media with effusion, and uncommon ear diseases. However, duration and severity of ear infections were not reported.

The main outcome variable used in this study was *hearing problems* as reported by parent (“Does child have any of these ongoing problems… hearing problems (yes, no)?”). There was no information on cause, duration, and severity of hearing problems. Ear infections and hearing problems were asked with identical questions between waves. However, there were omitted questions on hearing problems in wave 2 of the B and K cohort and on ear infection in wave 3 of the K cohort.

In view of existing literature on the high prevalence on ear infections among indigenous children, separate analyses and results are reported for non-indigenous and indigenous children [31-33]. Confounder variables were sex, employment, and school completion of parents, remoteness, and SEIFA economic resource. Results present ear infections at baseline for all children with data. For subsequent analysis we exclude children with hearing problems at baseline in order to establish longitudinal impacts of ear infection on hearing impairment. Multivariate logistic regressions estimate the adjusted odds ratios and 95% confidence intervals of the associations between reported ear infections and reported hearing problems, all analyses used Stata version 12.

### Results

Reported ear infection and hearing problems during the 6-year follow-up were summarised in Table 2 (taking into account that some questions in selected waves had been omitted). Among the B cohort, reported ear infections of indigenous children were more than double compared to non-indigenous children between 0/1 to 4/5 years. Hearing problems were also more commonly reported among indigenous children in both B and K cohorts. Table 3 presented adjusted odds ratios between reported ear infection in preceding wave and subsequent ear infections between wave adjusting for possible confounders in Table 1 and stratified by indigenous status. Among the K cohort, strong associations were found between reported ear infection in preceding and subsequent wave after adjusting for possible confounders: AORs ranged from 6.5 to 17.7 among non-indigenous children and 9.8 to 17.5 among indigenous children. A similar magnitude of association was found among the B cohort but some were not statistically significant.

**Table 2:** Percent reported ear infections and hearing problems by indigenous status

**Age (year)**	**Non indigenous children**			
	**Ear infection % (n)**		**Hearing problems % (n)**	
	**B cohort**	**K cohort**	**B cohort**	**K cohort**
0/1	3.7 (178)		0.7 (35)	
2/3	5.4 (237)		n/a*	
4/5	5.5 (232)	7.7 (368)	2.0 (83)	3.1 (150)
6/7	5.0 (205)	5.6 (235)	2.8 (113)	n/a
8/9		n/a		2.5 (105)
10/11		3.2 (130)		2.1 (86)
**Age (year)**	**Indigenous children**			
	**Ear infection % (n)**		**Hearing problems % (n)**	
	**B cohort**	**K cohort**	**B cohort**	**K cohort**
0/1	9.6 (22)		0.9 (2)	
2/3	9.4 (17)		n/a*	
4/5	8.1 (12)	13.9 (26)	4.0 (6)	7.5 (14)
6/7	4.8 (7)	5.9 (9)	4.8 (7)	n/a
8/9		n/a		4.0 (5)
10/11		7.6 (9)		2.5 (3)

*n/a: data not available.

**Table 3:** Longitudinal analyses of ear infection in preceding wave and subsequent ear infections by indigenous status

		Non-indigenous children - AOR [95% CI] ear infections between wave*			
		**2/3 years**	**4/5 years**	**6/7 years**	**10/11 years**
**B cohort**					
0/1	No	ref	ref	ref	
	Yes	**7.6 [5.1-11.3]**	**4.6 [2.9-7.4**]	**1.9 [1.0-3.7]**	
2/3	No		ref	ref	
	Yes		**8.0 [5.5-11.6]**	**4.1 [2.5-6.8]**	
4/5	No			ref	
	Yes			**12.3 [8.0-18.9]**	
**K cohort**					
4/5	No			ref	ref
	Yes			**9.3 [6.8-12.6]**	**6.5 [4.2-9.9]**
6/7	No				ref
	Yes				**17.7 [11.2-26.4]**
		Indigenous children - AOR [95% CI] reported ear infections between wave*			
		**2/3 years**	**4/5 years**	**6/7 years**	**10/11 years**
**B cohort**					
0/1	No	ref	ref	ref	
	Yes	2.8 [0.6-13.1]	**4.7 [1.1-20.5**]	1.4 [0.09-22.4]	
2/3	No		ref	ref	
	Yes		**9.4 [1.7-51.8]**	3.4 [0.39-29.7]	
4/5	No			ref	
	Yes			**25.3 [4.6-139.7]**	
**K cohort**					
4/5	No			ref	ref
	Yes			**9.8 [1.6-59.3]**	**10.1 [2.4-42.9]**
6/7	No				ref
	Yes				**17.5 [1.1-287.9]**

* Adjusted Odds Ratios (AOR) and 95% Confidence Interval [95% CI]; Confounder variables were sex, employment and school completion of parents, remoteness, and SEIFA economic resource (shown in Table 1).

Longitudinal associations between ear infections and hearing problems (excluding hearing problems at baseline) were reported in Table 4. Among non-indigenous children in the B cohort, hearing problems at age 6/7 years were associated with reporting ear infection at preceding waves (AORs 2.6, 3.9, 5.2 aged 0/1, 2/3, and 4/5 years), respectively. Among non-indigenous children in the K cohort, hearing problems at age 8/9 and 10/11 years were associated with reporting ear infection at age 4/5 years (AOR 4.0, 95% CI 2.2-7.3 and AOR 3.9, 95% CI 2.1-7.4). Corresponding AORs among indigenous children were 7.7 (95% CI 1.0-59.4) and 15.7 (95% CI 1.3-186.0). In many cases, there was no observation for indigenous children due to small samples.

**Table 4:** Longitudinal analyses of ear infection and hearing problems in subsequent waves by indigenous status (excluding hearing impairment at baseline)

		Non-indigenous children - AOR [95% CI] ear infections and hearing problems*			
		**4/5 years**	**6/7 years**	**8/9 years**	**10/11 years**
**B cohort**					
**0/1**	No	ref	ref		
	Yes	2.3 [0.9-6.0]	**2.6 [1.2-5.7]**		
**2/3**	No	ref	ref		
	Yes	**2.5 [1.2-5.1]**	**3.9 [2.1-7.6]**		
**4/5**	No		ref		
	Yes		**5.2 [2.9-9.4]**		
**K cohort**					
**4/5**	No			ref	ref
	Yes			**4.0 [2.2-7.3]**	**3.9 [2.1-7.4]**
**6/7**	No			ref	ref
	Yes			**9.8 [5.6-17.1]**	**10.2 [5.4-19.2]**
		Indigenous children - AOR [95% CI] ear infections and hearing problems*			
		**4/5 years**	**6/7 years**	**8/9 years**	**10/11 years**
**B cohort**					
**0/1**	No		ref		
	Yes	(no observation)	2.0 [0.2-20.7]		
**2/3**	No		ref		
	Yes	(no observation)	(no observation)		
**4/5**	No		ref		
	Yes		6.1 [0.8-46.9]		
**K cohort**					
**4/5**	No			ref	ref
	Yes			**7.7 [1.0-59.4]**	**15.7 [1.3-186.0]**
**6/7**	No				
	Yes			(no observation)	(no observation)

* Adjusted Odds Ratios (AOR) and 95% Confidence Interval [95% CI]; Confounder variables were sex, employment and school completion of parents, remoteness, and SEIFA economic resource (shown in Table 1).

Longitudinal analyses of repeated ear infection and hearing problems during the 6-year follow up (Table 5) were: among non-indigenous children, comparing to those never reported ear infection, the association between number of repeated ear infections and hearing problems at age 6/7 years increased substantially and statistically significantly in both B and K cohorts (AORs ranged from 4.5 to 31.7 and 4.6 to 51.0, respectively). However, these associations were not statistically significant among indigenous children in both cohorts.

**Table 5:** Longitudinal analyses of ear infections and hearing problems by indigenous status (excluding hearing impairment at baseline)*

**Non-indigenous children**					
**B cohort**			**K cohort**		
Number of ear infections	% (n)	Hearing problems at age 6/7 years AOR [95% CI]	Number of ear infections	% (n)	Hearing problems at age 10/11 years AOR [95% CI]
Never		ref	Never		ref
Once	9.6 (462)	**4.5** [2.8-7.1]	Once	8.7 (402)	**4.6** [2.4-8.7]
Twice	2.8 (137)	**8.9** [4.9-15.9]	Twice	1.6 (72)	**17.1** [7.9-36.7]
Three times +	0.5 (24)	**31.7** [12.7-79.3]	Three times +	0.5 (22)	**51.0** [19.2-135.4]
**Indigenous children**					
**B cohort**			**K cohort**		
Number of ear infections	% (n)	Hearing problems at age 6/7 years AOR [95% CI]	Number of ear infections	% (n)	Hearing problems at age 10/11 years AOR [95% CI]
Never		ref	Never		ref
Once	13.9 (31)	n/a	Once	11.6 (20)	6.1 [0.3-127.0]
Twice	4.8 (11)	4.8 [0.6-34.2]	Twice	2.9 (5)	41.9 [0.9-1958.6]
Three times +	0.4 (1)	n/a	Three times +	1.2 (2)	n/a

* Adjusted Odds Ratios (AOR) and 95% Confidence Interval [95% CI]; Confounder variables were sex, employment and school completion of parents, remoteness, and SEIFA economic resource (shown in Table 1).

## Discussion

This paper examines the epidemiology of ear infection and hearing problems among two cohorts of Australian children. By using 6-year prospective cohort data, we established the longitudinal impacts of ear infection on hearing problems at various ages. We also found that repeated ear infection was substantially and significantly associated with hearing problems at later age.

Our findings support an earlier study on impact of middle ear disease associated with hearing loss [34]. As well, we confirm that the onset of ear infection is associated with an increased likelihood of repeated episodes and these children are at a much higher risk of long-term negative impact [35]. Our findings provide clear evidence that ear infection leads to hearing impairment and the consequences of ear infections may persist throughout early childhood, potentially compromising children’s language acquisition, learning ability, and social interactions [36].

Findings on the sequelae of repeated ear infection and subsequent hearing problems were both striking and concerning. A multi-centre cross-sectional study among children in the US reported correlation between frequency of otitis media, worsening overall physical health, and impact on caregivers’ time and emotional concerns [15]. Our data on repeated ear infection between waves have shown that follow-up is necessary for children with repeated ear infection episodes if the problems persist [37,38].

The strength of this study is the use of the Longitudinal Study of Australian Children, a nationally representative study that includes a wide array of baseline and repeated exposures and outcomes. A large sample size allows sufficient statistical power to examine multiple episodes of ear infections and longitudinal design allows assessment of impact over time [26]. We acknowledge the possible limitation of the questions and the parent-reported nature of self-reported ear infections on behalf of children and could include various symptoms. We also note that the question related to ear infections only allows a ‘yes-no’ response, which could only capture a maximum of one episode a year. This may underreport the magnitude of ear infections and do not take into account repeated ear infection episodes within the same year. A recent population-based study in Scandinavia has shown self-reported otitis media to be relatively reliable and suggested that any inconsistency in reporting is likely to be associated with less severe episodes [39]. As well, we also acknowledge the possible limitation of parent-reported hearing impairment with severity not known. However, another cross-sectional study based on the Longitudinal Study of Children Survey of cohort aged 4 to 5 years has used this hearing variable and reported association between hearing impairment and language development, educational outcome, and metal health in children [14].

## Conclusion

This paper makes a contribution to the limited longitudinal evidence to date on the impact of ear infection on subsequent hearing problems in both indigenous and non-indigenous children. Our findings highlight the need for follow-up services and strategies to minimise substantial, long-term impacts on hearing associated with ear infection in early childhood.

## Abbreviations

LSAC: Longitudinal Study of Australian Children; SEIFA: Socio-Economic Indexes for Areas; B cohort: Baby cohort; K cohort: Kindergarten cohort; AOR: Adjusted Odds Ratio; CI: Confidence Interval

## Competing interests

This work was supported by an unconditional grant from the GlaxoSmithKline. The authors declare that they have no competing interests.

## Authors’ contributions

VY and AH conceptualized and designed the study. VY analysed data and drafted the manuscript. AH and LS provided comments on the initial manuscript and subsequent revisions. All authors approved the final manuscript.

## Authors’ information

Based at the Australian National University, VY and LS are researchers at the National Centre for Epidemiology and Population and AH is working at the School of Sociology.

## Pre-publication history

The pre-publication history for this paper can be accessed here:

http://www.biomedcentral.com/1471-2431/13/28/prepub

## References

[B1] Morris PS Leach AJ Acute and chronic otitis media Pediatr Clin North Am 2009 56 6 1383 1399 10.1016/j.pcl.2009.09.007 19962027 PMC7111681

[B2] Rovers MM Schilder AG Zielhuis GA Rosenfeld RM Otitis media Lancet 2004 363 9407 465 473 10.1016/S0140-6736(04)15495-0 14962529

[B3] Pirozzo S Del Mar C Acute otitis media West J Med 2001 175 6 402 407 10.1136/ewjm.175.6.402 11733434 PMC1275975

[B4] Elden LM Spiro DM Acute otitis media-to treat or not to treat? Pediatr Emerg Care 2006 22 4 283 286 10.1097/01.pec.0000217663.19639.2d 16651926

[B5] Schloss MD Otitis media: to treat or not to treat? Can Respir J 1999 6 Suppl A 51A 53A 10202235

[B6] Stevanovic T Komazec Z Lemajic-Komazec S Jovic R Acute otitis media: to follow-up or treat? Int J Pediatr Otorhinolaryngol 2010 74 8 930 933 10.1016/j.ijporl.2010.05.017 20599127

[B7] Karevold G Kvestad E Nafstad P Kvaerner KJ Respiratory infections in schoolchildren: co-morbidity and risk factors Arch Dis Child 2006 91 5 391 395 10.1136/adc.2005.083881 16464964 PMC2082748

[B8] Morris PS Upper respiratory tract infections (including otitis media) Pediatr Clin North Am 2009 56 1 101 117 x 10.1016/j.pcl.2008.10.009 19135583 PMC7118466

[B9] Davidson J Hyde ML Alberti PW Epidemiology of hearing impairment in childhood Scand Audiol Suppl 1988 30 13 20 3227256

[B10] Clements DA Otitis media and hearing loss in a small aboriginal community Med J Aust 1968 1 16 665 667 5653322 10.5694/j.1326-5377.1968.tb29015.x

[B11] Shriberg LD Friel-Patti S Flipsen P Jr Brown RL Otitis media, fluctuant hearing loss, and speech-language outcomes: a preliminary structural equation model J Speech Lang Hear Res 2000 43 1 100 120 10668655 10.1044/jslhr.4301.100

[B12] Roberts JE Rosenfeld RM Zeisel SA Otitis media and speech and language: a meta-analysis of prospective studies Pediatrics 2004 113 3 Pt 1 e238 e248 14993583 10.1542/peds.113.3.e238

[B13] Thorne JA Middle ear problems in Aboriginal school children cause developmental and educational concerns Contemp Nurse 2003 16 1–2 145 150 14994905 10.5172/conu.16.1-2.145

[B14] Hogan A Shipley M Strazdins L Purcell A Baker E Communication and behavioural disorders among children with hearing loss increases risk of mental health disorders Aust N Z J Public Health 2011 35 4 377 383 10.1111/j.1753-6405.2011.00744.x 21806734

[B15] Lee J Witsell DL Dolor RJ Stinnett S Hannley M Quality of life of patients with otitis media and caregivers: a multicenter study Laryngoscope 2006 116 10 1798 1804 10.1097/01.mlg.0000231305.43180.f6 17003729

[B16] Brouwer CN Rovers MM Maille AR Veenhoven RH Grobbee DE Sanders EA Schilder AG The impact of recurrent acute otitis media on the quality of life of children and their caregivers Clin Otolaryngol 2005 30 3 258 265 10.1111/j.1365-2273.2005.00995.x 16111423

[B17] Access Economics The cost burden of otitis media in Australia 2009 Perth: GlaxoSmithKline

[B18] Taylor PS Faeth I Marks MK Del Mar CB Skull SA Pezzullo ML Havyatt SM Coates HL Cost of treating otitis media in Australia Expert Rev Pharmacoecon Outcomes Res 2009 9 2 133 141 10.1586/erp.09.6 19402800

[B19] Klein JO The burden of otitis media Vaccine 2000 19 Suppl 1 S2 S8 11163456 10.1016/s0264-410x(00)00271-1

[B20] Gunasekera H Knox S Morris P Britt H McIntyre P Craig JC The spectrum and management of otitis media in Australian indigenous and nonindigenous children: a national study Pediatr Infect Dis J 2007 26 8 689 692 10.1097/INF.0b013e3180621177 17848879

[B21] Coates HL Morris PS Leach AJ Couzos S Otitis media in Aboriginal children: tackling a major health problem Med J Aust 2002 177 4 177 178 12175319 10.5694/j.1326-5377.2002.tb04727.x

[B22] Gibney KB Morris PS Carapetis JR Skull SA Smith-Vaughan HC Stubbs E Leach AJ The clinical course of acute otitis media in high-risk Australian Aboriginal children: a longitudinal study BMC Pediatr 2005 5 1 16 10.1186/1471-2431-5-16 15955251 PMC1177962

[B23] O'Connor TE Perry CF Lannigan FJ Complications of otitis media in Indigenous and non-Indigenous children Med J Aust 2009 191 9 Suppl S60 S64 19883359 10.5694/j.1326-5377.2009.tb02929.x

[B24] Sanson A Nicholson J Ungerer J Zubrick S Wilson K Ainsley J Berthelsen D Bittman M Broom D Harrison L Introducing the Longitudinal Study of Australian Children, LSAC Discussion Paper No.1 2002 In. Melbourne: Australian Institute of Family Studies

[B25] Gray M Smart D Growing Up in Australia: The Longitudinal Study of Australian Children is now walking and talking Family Matters 2008 79 5 13

[B26] Nicholson JM Sanson A A new longitudinal study of the health and wellbeing of Australian children: how will it help? Med J Aust 2003 178 6 282 284 12633487 10.5694/j.1326-5377.2003.tb05197.x

[B27] Sanson A Misson S Wake M Zubrick S Silburn S Rothman S Dickenson J Summarising children’s wellbeing: the LSAC Outcome Index. LSAC Technical Paper No. 2 2005 In. Melbourne: Australian Institute of Family Studies

[B28] Soloff C Lawrence D Johnstone R Sample design. LSAC Technical Paper No. 1 2005 In. Melbourne: Australian Institute of Family Studies

[B29] Soloff C Lawrence D Sebastian M Johnstone R Wave 1 weighting and non-response. LSAC Technical Paper No. 3 2006 In. Melbourne: Australian Institute of Family Studies

[B30] ABS Information Paper: An Introduction to Socio-Economic Indexes for Areas (SEIFA) ABS Catalogue No. 2039.0 2006 Commonwealth of Australia: Australian Bureau of Statistics

[B31] Leach AJ Otitis media in Australian Aboriginal children: an overview Int J Pediatr Otorhinolaryngol 1999 49 Suppl 1 S173 S178 10577800 10.1016/s0165-5876(99)00156-1

[B32] Leach AJ Morris PS Perspectives on infective ear disease in indigenous Australian children J Paediatr Child Health 2001 37 6 529 530 10.1046/j.1440-1754.2001.00729.x 11903828

[B33] Leach AJ Morris PS The burden and outcome of respiratory tract infection in Australian and aboriginal children Pediatr Infect Dis J 2007 26 10 Suppl S4 S7 18049380 10.1097/INF.0b013e318154b238

[B34] Williams CJ Coates HL Pascoe EM Axford Y Nannup I Middle ear disease in Aboriginal children in Perth: analysis of hearing screening data, 1998–2004 Med J Aust 2009 190 10 598 600 19450214 10.5694/j.1326-5377.2009.tb02576.x

[B35] Williams CJ Jacobs AM The impact of otitis media on cognitive and educational outcomes Med J Aust 2009 191 9 Suppl S69 S72 19883361 10.5694/j.1326-5377.2009.tb02931.x

[B36] O'Leary SJ Triolo RD Surgery for otitis media among Indigenous Australians Med J Aust 2009 191 9 Suppl S65 S68 19883360 10.5694/j.1326-5377.2009.tb02930.x

[B37] Monobe H Ishibashi T Fujishiro Y Shinogami M Yano J Factors associated with poor outcome in children with acute otitis media Acta Otolaryngol 2003 123 5 564 568 12875576

[B38] Pichichero ME Recurrent and persistent otitis media Pediatr Infect Dis J 2000 19 9 911 916 10.1097/00006454-200009000-00034 11001126

[B39] Kvestad E Kvaerner KJ Roysamb E Tambs K Harris JR Magnus P The reliability of self-reported childhood otitis media by adults Int J Pediatr Otorhinolaryngol 2006 70 4 597 602 10.1016/j.ijporl.2005.08.005 16143406

